# Genome-wide association analysis of treatment resistant schizophrenia for variant discovery and polygenic assessment

**DOI:** 10.1186/s40246-024-00673-x

**Published:** 2024-09-27

**Authors:** Hasan Çağın Lenk, Elise Koch, Kevin S. O’Connell, Robert Løvsletten Smith, Ibrahim A. Akkouh, Srdjan Djurovic, Ole A. Andreassen, Espen Molden

**Affiliations:** 1 Center for Psychopharmacology, Diakonhjemmet Hospital, Oslo, Norway; 2 Section for Pharmacology and Pharmaceutical Biosciences, Department of Pharmacy, University of Oslo, Oslo, Norway; 3 Centre for Precision Psychiatry, Centre for Mental Disorders Research, Division of Mental Health and Addiction, Oslo University Hospital, and Institute of Clinical Medicine, University of Oslo, Oslo, Norway; 4 Department of Medical Genetics, Oslo University Hospital, Oslo, Norway; 5 KG Jebsen Centre for Neurodevelopmental Disorders, University of Oslo and Oslo University Hospital, Oslo, Norway

## Abstract

**Background:**

Treatment resistant schizophrenia (TRS) is broadly defined as inadequate response to adequate treatment and is associated with a substantial increase in disease burden. Clozapine is the only approved treatment for TRS, showing superior clinical effect on overall symptomatology compared to other drugs, and is the prototype of atypical antipsychotics. Risperidone, another atypical antipsychotic with a more distinctive dopamine 2 antagonism, is commonly used in treatment of schizophrenia. Here, we conducted a genome-wide association study on patients treated with clozapine (TRS) vs. risperidone (non-TRS) and investigated whether single variants and/or polygenic risk score for schizophrenia are associated with TRS status. We hypothesized that patients who are treated with clozapine and risperidone might exhibit distinct neurobiological phenotypes that match pharmacological profiles of these drugs and can be explained by genetic differences. The study population (*n* = 1286) was recruited from a routine therapeutic drug monitoring (TDM) service between 2005 and 2022. History of a detectable serum concentration of clozapine and risperidone (without TDM history of clozapine) defined the TRS (*n* = 478) and non-TRS (*n* = 808) group, respectively.

**Results:**

We identified a suggestive association between TRS and a common variant within the *LINC00523* gene with a significance just below the genome-wide threshold (*rs79229764 C* > *T*, OR = 4.89; *p* = 1.8 × 10^−7^). Polygenic risk score for schizophrenia was significantly associated with TRS (OR = 1.4, *p* = 2.1 × 10^−6^). In a large post-mortem brain sample from schizophrenia donors (n = 214; CommonMind Consortium), gene expression analysis indicated that the *rs79229764* variant allele might be involved in the regulation of *GPR88* and *PUDP*, which plays a role in striatal neurotransmission and intellectual disability, respectively.

**Conclusions:**

We report a suggestive genetic association at the rs79229764 locus with TRS and show that genetic liability for schizophrenia is positively associated with TRS. These results suggest a candidate locus for future follow-up studies to elucidate the molecular underpinnings of TRS. Our findings further demonstrate the value of both single variant and polygenic association analyses for TRS prediction.

**Supplementary Information:**

The online version contains supplementary material available at 10.1186/s40246-024-00673-x.

## Introduction

Antipsychotics is the cornerstone of schizophrenia treatment, but up to 30% of patients do not respond adequately to standard treatment [1]. These treatment-resistant schizophrenia (TRS) patients suffer from greater symptom burden [1], and clozapine is the only evidence-based treatment for TRS with superior clinical effect on overall positive and negative symptoms [2].

All atypical antipsychotics broadly share antagonism at dopamine 2 (D_2_) and serotonin 2A (5-HT_2A_) receptors, but they also have binding capabilities at other receptors [3]. Clozapine has transient and low affinity binding at D_2_ receptors while favouring antagonism on 5-HT_2A_ receptors [3]. It exhibits the broadest coverage of molecular targets among all antipsychotics, including modulation of glutaminergic, cholinergic, and noradrenergic transmission [3]. Risperidone is a commonly used atypical antipsychotic with a more distinct receptor profile and particularly stronger D_2_ antagonism than clozapine [3]. Patients who respond adequately to antipsychotics like risperidone are considered as ‘responders’ to D_2_ receptor antagonism. Since clozapine can only be prescribed after failed response to two other antipsychotics, those who use clozapine may be considered as ‘non-responders’, meaning that symptom improvement is not sufficient with antagonism of these receptors but may require involvement of other neurotransmitter systems.

Currently, knowledge on the underlying neurobiology of TRS is limited. While emerging evidence suggests that TRS has a polygenic architecture [4], previous studies have failed to identify specific gene variants that robustly correlate with TRS [5]. In a genome-wide investigation, Ruderfer et al. identified shared genetic underpinnings between schizophrenia pathophysiology and the mechanisms of action of antipsychotic drugs [6]. Here, we conducted a genome-wide association study (GWAS) on clozapine use, defined as proxy of resistant schizophrenia, vs. risperidone use, defined as proxy for responsive schizophrenia, in patients with confirmed therapeutic concentrations of these two drugs. We both analysed for associations with single variants and polygenic risk score for schizophrenia and hypothesized that genetic differences between patients treated with clozapine and risperidone might reflect aspects underlying the genetic architecture of antipsychotic response.

## Methods

### Study population

In clinical practice, therapeutic drug monitoring (TDM) may be applied to guide antipsychotic dosing to ensure sufficient treatment intensity, and thus exclude potential ‘pseudo resistance’ and erroneous diagnosis of TRS. The study utilized retrospective data/samples of patients from the routine TDM service at Center for Psychopharmacology, Diakonhjemmet Hospital, Oslo, Norway, who in the period between January 2005 and August 2022 had performed (i) TDM of clozapine and/or risperidone and (ii) genotyping of cytochrome P450 (CYP) enzymes. In Norway, TDM is reimbursed and commonly used as a clinical tool in public psychiatric health service to assess adherence to medication and optimize dosing of drugs for maximizing clinical effect and/or minimizing side effects. Use of *CYP* genotyping is also reimbursed and increasingly used to guide dose optimization of psychiatric drugs.

The study population consisted of patients of Norwegian inhabitants and is assumed to be of patients with Caucasian ancestry. The main inclusion criterion was the availability of biobanked (residual) blood samples from CYP genotyping and clinical concentration levels of clozapine or risperidone verified by TDM. Patients were phenotyped based on their history of using clozapine or risperidone at clinically relevant concentrations, as determined by their TDM history. The TRS group was defined by clozapine use, while the non-TRS group was defined by risperidone use without any history of clozapine TDM. Further requirements for being included in the risperidone cohort were treatment with ≥ 2 mg/day risperidone at least once (dose range for psychotic disorders) and no previous use/TDM history of clozapine.

In the non-TRS group, 41% of the patients had a TDM history of antipsychotic polypharmacy with risperidone (two or more drugs). Furthermore, in the non-TRS group, TDM history of median number of other, non-clozapine antipsychotics before taking risperidone was 1 (IQR: 0,1), whereas in the clozapine (TRS) group, TDM history of median number of non-clozapine antipsychotics was 2 (IQR; 0,3). All patients were included in the study independent of the clinical setting (out-/inpatient). In Norway, TDM of clozapine is usually performed for dose titration and by routine during ongoing treatment, whereas TDM of risperidone is more often ‘reactive’ due to insufficient clinical effect or suspected side effects. Center for Psychopharmacology is the major laboratory performing the analyses.

The Regional Committee for Medical and Health Research Ethics and the Investigational Review Board at Diakonhjemmet Hospital approved the study. The study used anonymized data and residual blood samples from already performed routine analyses. In these cases, where study inclusion does not pose any burden to the patients, the Health Research Act of Norway allows for using samples and information collecting in clinical routine for research purpose without written informed patient consent. However, it is mandatory to send information to the patients, in advance of starting a project, about their legal rights to reserve against being included. Accordingly, letters were sent to all patients eligible for inclusion, where those requesting to opt-out were excluded from the study.

### Serum concentration determination of antipsychotics

Liquid chromatography with mass spectrometry (LC–MS) method with FDA certified and validated analytical assays were applied for determination of serum concentrations of clozapine and risperidone and their metabolites. Lower limit of quantification for clozapine and *N*-desmethylclozapine was 50 nmol/L, whereas this limit was 1 nmol/L and 2 nmol/L for risperidone and 9-hydroxyrisperidone, respectively.

Serum concentration thresholds, based on consensus guidelines [7], were used as an informative measure to assess whether exposure to antipsychotic was adequate. These were clozapine 1070 nmol/L (350 µg/L) and risperidone active moiety 50 nmol/L (20 µg/L, risperidone plus 9-hydroxyrisperidone).

### Genotyping and imputation

As previously described in detail [8], DNA extracted from the whole blood was genotyped using the Human Omni Express-24 v.1.1 (Illumina Inc., San Diego, CA, USA), at deCODE Genetics (Reykjavik, Iceland), following standard Illumina protocols. Quality control and phasing of chromosome-wide haplotypes were performed with PLINK v1.93 [9, 10] and Eagle2 [11, 12], respectively. The first release of the haplotype reference consortium reference set was used for imputation of missing variants with Minimac3 [13]. Following imputation, exclusion criteria were variants with (1) minor allele frequency < 1% or (2) departure from Hardy–Weinberg equilibrium (*P* value < 1 × 10^−6^), or individuals with (3) high rates of missing genotypes (> 5%), that exhibit relatedness (pairwise Identity-By-Descent ^ π > 0.2 according to PLINK v1.9). 1286 individuals with ~ 5.6 million common variants were included for further statistical analysis.

### Statistical analysis

The statistical analyses were performed using R statistics [14] for demographic characteristics of the study sample and follow-up analysis of the lead variant identified. Distribution of sex and age were compared between TRS and non-TRS patients using Pearson’s Chi-squared test and Welch’s two sample t-test, respectively. If risperidone patients were prescribed long-acting injectable formulations, total daily dosing was estimated by dividing the prescribed doses by dosing intervals. Estimated means of serum concentrations (nmol/L) and daily dose (mg/day) were calculated using linear mixed models with unique patient identifier as random effects.

The GWAS of TRS versus non-TRS was conducted using logistic regression analyses implemented in PLINK v1.9 [9, 10], controlling for participant age, sex, the first 10 genetic principal components and genotyping batch. Standard GWAS threshold (5 × 10^−8^) was used to define statistical significance of the GWAS analysis. LDproxy module provided by the LDlink platform [15] was used to perform linkage equilibrium analysis of the lead variant.

For the expression quantitative trait loci (eQTL) analysis, genotype and post-mortem frontal brain cortex gene expression data were obtained from the CommonMind Consortium [16]. Only data from schizophrenia patients with European ethnicity (n = 214) were included in the study. Genotype QC and imputation were performed by the Consortium. The expression data was filtered and normalized using the DESeq2 R package [17]. The R package MatrixEQTL [18] was used for the cis-eQTL analysis, adjusting for age, gender and post-mortem interval. Variant-gene associations within 1 Mb of the identified lead variant were considered.* P* < 0.05 defined statistical significance of patient/treatment characteristics and gene expression analyses.

For the GWAS sample, a schizophrenia polygenic risk score (PRS) was calculated based on the latest schizophrenia GWAS performed by the PGC [19] using the meta-analysis of European samples comprising 53,386 cases and 77,258 controls. The PRS was calculated using the PRS continuous shrinkage (PRS-cs) approach [20], adjusting for linkage disequilibrium (LD) based on the LD structure of the European sample of the 1000 Genomes Phase III [21] with default options and a shrinkage parameter of phi = 1 [20]. To facilitate the interpretability of the results, the PRS was standardized (mean = 0, SD = 1) before statistical analysis. To investigate if the PRS for schizophrenia as well as smoking, age, and sex is associated with TRS, a logistic regression analysis including TRS (yes/no) as the dependent variable was performed. The model included the PRS, age, sex, smoking (yes/no), as well as genotyping batch and the first 5 principal components for genetic ancestry for adjustment.

## Results

Sample characteristics are presented in Table 1. The study population (n = 1286) included n = 478 TRS patients and n = 808 non-TRS patients. There was a higher proportion of males among the TRS group compared to the non-TRS group (59% vs 53%, *p* = 0.009), but no difference in age distribution (Table 1). Mean daily dose estimates were 335 and 3.3 mg/day for patients who were treated with clozapine (TRS) and risperidone (non-TRS), respectively (Table 1). Furthermore, estimated serum concentration measurements for patients in the TRS and non-TRS group were 1201 nmol/L (393 ng/ml) for clozapine and 64 nmol/L for risperidone (active moiety including 9-hydroxyrisperidone), respectively (Table 1). Among the TRS group, median number of clozapine measurements during the study period was 14 (IQR: 5, 36) spanning 4.3 years (IQR: 0.7, 11.8) of TDM, whereas TDM history of the non-TRS group showed median of 3 (IQR: 1.8, 7) risperidone measurements over 0.8 years (IQR: 0.04, 4.8; Table 1).

**Table 1:** Demographic and treatment characteristics of the study population

Variables	TRS	non-TRS	*p*
Number of patients, *n*	478	808	–
Male, *n* (%)	284 (59)	430 (53)	0.031
Age, years; mean (SD)	48.7 (15.0)	48.7 (16.9)	0.99
Serum concentration, nmol/L; mean (95%CI)	1201 (1162,1240)	64 (30, 98)	–
Dose, mg/day; mean (95%CI)	335 (320, 350)	3.3 (3.2, 3.4)	–
History of TDM measurements, *n*; median (IQR)	14 (5, 36)	3 (1.8, 7)	–
TDM duration, years; median (IQR)	4.3 (0.7, 11.8)	0.8 (0.04, 4.8)	–

*IQR* Interquartile range, *SD* Standard deviation, *TDM* Therapeutic drug monitoring, *TRS* treatment resistant schizophrenia. TRS and non-TRS groups comprised of patients who were treated with clozapine and risperidone, respectively

**Fig. 1:**
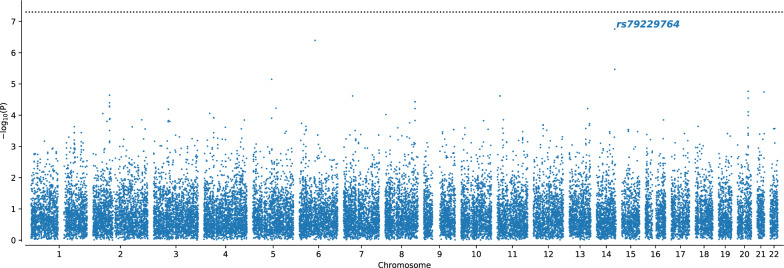
Manhattan plot showing the associations with risk of TRS. The − log10 transformed *p*-values for each SNP are shown on the y-axis and chromosomal positions are indicated on the x-axis. The dashed black line represents the standard GWAS p-value threshold of 5 × 10^−8^. The most significant SNP is highlighted by text

The GWAS on TRS did not show any associations reaching genome-wide significance (*p* < 5 × 10^−8^), however identified a single nucleotide polymorphism (SNP; *rs79229764 C* > *T*) on chromosome 14 with a *p*-value just above the genome-wide significance threshold (*p* = 1.8 × 10^−7^; Fig. 1). We observed a 2.1-fold higher proportion of the risk allele carriers among TRS patients compared to non-TRS patients with allele frequencies of 3.7% and 1.7%, respectively; OR = 4.89, 95% CI = [2.6, 8.5]). Frequency of the minor allele variant is 4.2% among European populations (https://www.ensembl.org/). LD structure from the 1000 Genomes phase 3-genome browser showed a low LD region (Supplementary Fig. 1) with variants that are in low to moderate LD with the *rs79229764* haplotype among the European populations (R2 < 0.54; Supplementary Table 1).

**Fig. 2:**
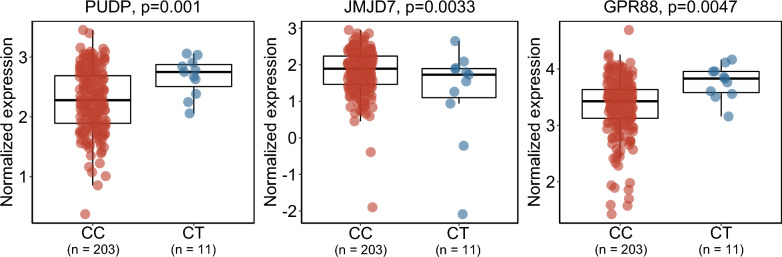
Impact of *rs79229764 C* > *T* on brain tissue gene expression from CommonMind Consortium data. The 3 most significant gene expression associations were illustrated. Normalized expression results are shown on the y-axis, while *rs79229764 C* > *T* genotypes are indicated on the x-axis. Gene names and unadjusted p-values from association analyses are shown at the top part of the plots

The *rs79229764* variant is located within the exon of the long intergenic non-coding RNA 523 (*LINC00523*) gene. NHGRI-EBI GWAS Catalog did not reveal any previous associations with the *rs79229764* variant (https://www.ebi.ac.uk/gwas/). Expression quantitative trait loci (eQTL) analysis of *rs79229764* using the CommonMind Consortium post-mortem brain expression data [16] from *n* = 214 (*CC*: *n* = 203, *CT*: *n* = 11) schizophrenia patients revealed gene associations with nominal significance. The top 3 most significant risk allele-gene relationships included increased expression of *PUDP* (*p* = 0.001) and *GPR88* (*p* = 0.0047), and decreased expression of *JMJD7* (*p* = 0.003; Table 2 and Fig. 2).

**Table 2:** The 3 most significant brain tissue gene expression associations of the lead variant *rs79229764 C* > *T* from CommonMind Consortium

Chromosome	Position	Gene name	Beta	*p* (unadjusted)*	Gene type
X	6,667,865–7,148,158	PUDP	0.441	0.001	Protein coding
15	41,828,092–41,837,581	JMJD7	-0.592	0.003	Protein coding
1	100,538,139–100,542,021	GPR88	0.435	0.005	Protein coding

Betas are included from the eQTL analysis which was adjusted for age, gender and post-mortem interval. Only data from schizophrenia patients with European ethnicity were included in the analysis. * *p* > 0.05 after multiple comparisons corrections

**Fig. 3:**
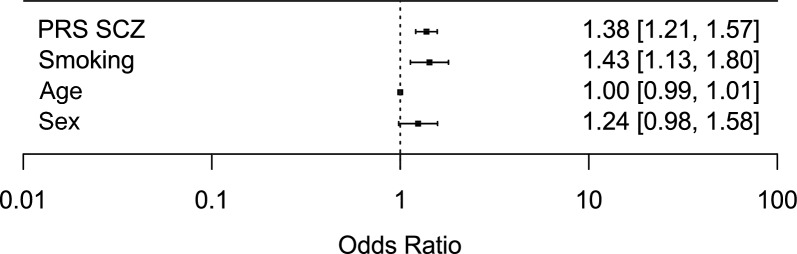
Forest plot showing the association between treatment resistant schizophrenia and schizophrenia polygenic risk score (PRS SCZ), smoking, age, and sex. Effects are reported as odds ratios (95% confidence interval)

The schizophrenia PRS showed a significant, positive association with TRS, i.e., higher PRS was associated with being a TRS case (OR = 1.4, *p* = 2.1 × 10^−6^; Fig. 3 and Supplementary Table 2). In addition, we observed that smoking was significantly associated with TRS (OR = 1.4, *p* = 0.003; Fig. 3 and Supplementary Table 2). No association was observed between TRS and sex or age (Fig. 3 and Supplementary Table 2).

## Discussion

In the current study, we conducted a genome-wide investigation of TRS based on treatment history of clozapine (TRS) and risperidone (without clozapine history; non-TRS) at therapeutic serum concentrations in a well-phenotyped study cohort (*n* = 1286). We found that genetic liability to schizophrenia, as measured by schizophrenia PRS was significantly associated with TRS. We also identified a suggestive variant in the *LINC00523* gene (*rs79229764 C* > *T)* with strong enrichment in clozapine vs. risperidone patients (OR = 4.89) and a significance level close to the genome-wide threshold (*p* = 1.8 × 10^−7^). Follow-up eQTL analysis in post-mortem brain samples from schizophrenia donors (*n* = 214) suggested transcriptional effects of *rs79229764* on different genes.

Although previous studies investigating the association between schizophrenia PRS and TRS had conflicting results [22–25], our finding is in line with a recent systematic review and meta-analysis that showed significant associations between schizophrenia PRS and TRS [26]. We have previously observed that higher schizophrenia PRS is associated with antipsychotic prescription pattern [27, 28]. Together with the findings from the current study, these results suggest that schizophrenia PRS may have potential utility to aid in therapeutic decision making on antipsychotic treatment. Furthermore, this shows the ability of the study to use a combinatorial approach investigating pharmacogenomics of TRS at both overall and detailed levels.

Interestingly, tobacco smoking was also significantly associated with TRS. Approximately 60% of patients with schizophrenia are smokers [29, 30], and the “self-medication” hypothesis states that nicotine consumption via tobacco smoking reduces the negative and cognitive symptoms in patients with schizophrenia [31]. Supporting this hypothesis, among patients with TRS, smokers show worse cognitive performances and more severe negative symptoms compared to nonsmokers [32] which may be indicative of self-medication behaviour to alleviate these symptoms. While smoking is known to induce metabolism and reduce serum levels of clozapine, the present findings suggest that smoking of nicotine-containing products may be indicative of a higher disease burden, corresponding non-response to antipsychotics and TRS.

The *rs79229764* variant, identified as the closest to genome-wide significant association with TRS in our sample, is located in the exon of the *LINC00523* gene on the chromosome 14 open reading frame 70, C14orf70), which encodes an intergenic lncRNA (LINC00523). LINC00523 is highly expressed in basal ganglia (particularly in nucleus accumbens), testis, and adrenal gland (https://www.gtexportal.org/). Functional effect of the *rs79229764* variant on *LINC00523* expression is currently unknown, however LINC00523 has been previously shown to be downregulated in patients with type 2 diabetes mellitus (T2DM) [33]. It can be speculated that changes in the expression may be of clinical importance since T2DM, as well as tobacco smoking, are among the major risk factors of cardiovascular disease (CVD), which is reported to be the cause of death for 25% of patients with schizophrenia [34]. The contribution of unhealthy lifestyle [35] and metabolic adverse effects related to antipsychotic drugs (especially clozapine) [36] among patients with TRS are well-known, and current evidence also suggest that there may be a shared genetic liability between schizophrenia and CVD [37].

Investigation of the transcriptional effects of *rs79229764* variant in post-mortem brain data of schizophrenia patients suggested upregulation of *GRP88* and *PUDP*, and downregulation of *JMJD7*. Of particular interest may be the *GPR88* gene, which encodes an orphan G-protein coupled receptor of the class A rhodopsin family (GPR88) that has previously been suggested to be a candidate susceptibility gene for sporadic cases of schizophrenia and bipolar disorder [38, 39]. GPR88 is robustly expressed in the GABAergic medium spiny neurons (MSN) of the striatum [40–42] and central extended amygdala [43]. MSNs of the striatum are involved in dopaminergic and glutamatergic signalling pathways [44], which have been indicated in the pathophysiology of schizophrenia and treatment-resistance [45]. Furthermore, GPR88 expression has been previously shown to be altered after administration of mood stabilizers [46, 47], antidepressants [48–50], and drugs related to treatment of addiction [43] which highlights its relevance for the treatment of psychiatric disorders. Future studies should therefore elucidate the potential impact of the *rs79229764* polymorphism and/or GPR88 expression for the risk of TRS.

*PUPD* (or *HDHD1*) encodes a phosphatase which might be involved in RNA degradation [51]. Previous studies suggest involvement in cancer formation [52] and intellectual disability [53]. Associations with intellectual disability is in line with the latest evidence from the larger GWAS on TRS which showed polygenic correlations between TRS and poor cognitive performance and intelligence [4] and might indicate role of neurodevelopmental processes in formation of TRS [54].

One limitation of the current study is the relatively small sample size for a GWAS on complex traits such as TRS. To prevent concentration-dependent serious adverse effects and ensure adequate clinical exposure, TDM of clozapine is strongly recommended and performed more often compared to other antipsychotics. In the current study, this suggests that the clozapine-taking TRS group is more likely to represent general TRS population. On the other hand, since TDM of risperidone is usually performed on physician’s request, the risperidone taking non-TRS patients who are referred to TDM and pharmacogenetic investigation usually suffer from more serious psychiatric conditions and require closer follow-up. Therefore, non-TRS patients with gene variants associated with risk of treatment failure are likely to be overrepresented compared to general risperidone-taking patient population. Although not possible to quantify the magnitude of this effect, the high odds ratio of being carrier of the suggestive variant among clozapine-treated patients in our analysis may be underestimated. Another limitation is that we did not have access to clinical information of patients, which precluded a clinical assessment of treatment response status. However, the study utilizes longitudinal TDM data to include well-defined phenotypes of TRS and non-TRS patients where appropriate drug use was quantitatively evidenced by serum concentrations within the therapeutic ranges for the respective drugs [7]. With access to smoking status on the TDM requisition forms we were also able to identify and adjust for smoking as a predictor of TRS. Thus, the methodological approach in the current study facilitates use of robust phenotypes by accounting for smoking and eliminating pseudoresistance, an important confounding factor in TRS research, which may be driven by poor adherence or other factors [55]. Therefore, the strengths of the study, at least partially, may outweigh the above limitations.

## Conclusions

We conducted a GWAS of TRS that utilized a therapeutic drug monitoring patient sample, where smoking status was known, and therapeutic concentrations of clozapine and risperidone confirmed. Although the study did not reveal any genome-wide significant associations, we identified a variant in the *LINC0053* gene that was marginally associated with higher risk of TRS. Transcriptional effects of this variant suggest a role of striatal regulation and possible neurodevelopmental underpinnings in the pathophysiology of TRS. We also show that polygenic liability for schizophrenia was significantly associated with TRS. Our results suggest a candidate locus for future follow-up studies to elucidate the molecular underpinnings of TRS and demonstrate the value of combining single variant and polygenic association analyses to suggest potential utility of genetics in predicting TRS.

## Supplementary Information

Additional file1 (DOCX 196 KB)

## Data Availability

No datasets were generated or analysed during the current study.
