# Supplementary material for: Genome-wide association analysis of treatment resistant schizophrenia for variant discovery and polygenic assessment
Source: Hum Genomics. 2024 Sep 27;18:108. doi: 10.1186/s40246-024-00673-x (PMC11438281; doi:10.1186/s40246-024-00673-x)

# **Supplementary material**

**Genome-wide association analysis of treatment resistant schizophrenia for variant discovery and polygenetic assessment**

Hasan Çağın Lenk^1, 2, 3^, Elise Koch^3^, Kevin S. O’Connell^3^, Robert Løvsletten Smith^1^, Ibrahim A. Akkouh^3,4^, Srdjan Djurovic^3,4,5^, Ole A. Andreassen^3,5^, Espen Molden^1,2^

^1^ Center for Psychopharmacology, Diakonhjemmet Hospital, Oslo, Norway

^2^ Section for Pharmacology and Pharmaceutical Biosciences, Department of Pharmacy, University of Oslo, Oslo, Norway

^3^ Centre for Precision Psychiatry, Centre for Mental Disorders Research, Division of Mental Health and Addiction, Oslo University Hospital, and Institute of Clinical Medicine, University of Oslo, Oslo, Norway

^4^ Department of Medical Genetics, Oslo University Hospital, Oslo, Norway

^5^ KG Jebsen Centre for Neurodevelopmental disorders, University of Oslo and Oslo University Hospital, Oslo, Norway

**Supplementary Table 1.** Results of the linkage equilibrium analysis of the lead SNP *rs79229764 C>T.* Results of the SNPs with 25 highest R^2^ values are included in the table.

| SNP identifier | Coordinate | Alleles | MAF | R^2^ | Distance | Dprime | Correlated Alleles |
| --- | --- | --- | --- | --- | --- | --- | --- |
| rs79229764 | chr14:101138188 | (C/T) | 0.0417 | 1 | 0 | 1 | C=C,T=T |
| rs148266552 | chr14:101173495 | (CT/-) | 0.0408 | 0.5419 | 35307 | 0.7455 | C=CT,T=- |
| rs553064673 | chr14:101152069 | (T/C) | 0.0676 | 0.3805 | 13881 | 0.7957 | C=T,T=C |
| rs7144997 | chr14:101155432 | (A/G) | 0.0865 | 0.3693 | 17244 | 0.8957 | C=A,T=G |
| rs6575796 | chr14:101160363 | (A/G) | 0.0865 | 0.3693 | 22175 | 0.8957 | C=G,T=A |
| rs35799850 | chr14:101156850 | (C/T) | 0.0875 | 0.3646 | 18662 | 0.8956 | C=C,T=T |
| rs7152504 | chr14:101156924 | (G/A) | 0.0875 | 0.3646 | 18736 | 0.8956 | C=A,T=G |
| rs28421549 | chr14:101158232 | (C/T) | 0.0875 | 0.3646 | 20044 | 0.8956 | C=C,T=T |
| rs12880358 | chr14:101158809 | (C/G) | 0.0875 | 0.3646 | 20621 | 0.8956 | C=C,T=G |
| rs7142033 | chr14:101186169 | (A/G) | 0.0388 | 0.362 | 47981 | 0.6254 | C=A,T=G |
| rs7144576 | chr14:101186842 | (T/A) | 0.0348 | 0.3412 | 48654 | 0.6422 | C=T,T=A |
| rs12147845 | chr14:101144596 | (C/T) | 0.1143 | 0.3376 | 6408 | 1 | C=C,T=T |
| rs10130354 | chr14:101149511 | (T/C) | 0.0964 | 0.3268 | 11323 | 0.8946 | C=C,T=T |
| rs7144375 | chr14:101137384 | (T/C) | 0.1193 | 0.3217 | -804 | 1 | C=T,T=C |
| rs73358743 | chr14:101138506 | (C/T) | 0.1203 | 0.3187 | 318 | 1 | C=C,T=T |
| rs28478065 | chr14:101139651 | (C/A) | 0.1203 | 0.3187 | 1463 | 1 | C=C,T=A |
| rs17660362 | chr14:101136145 | (A/G) | 0.1203 | 0.3187 | -2043 | 1 | C=A,T=G |
| rs8014970 | chr14:101140792 | (T/G) | 0.1203 | 0.3187 | 2604 | 1 | C=T,T=G |
| rs17660210 | chr14:101131477 | (A/G) | 0.1203 | 0.3187 | -6711 | 1 | C=A,T=G |
| rs7156735 | chr14:101132229 | (T/C) | 0.1213 | 0.3157 | -5959 | 1 | C=T,T=C |
| rs8007901 | chr14:101130576 | (T/C) | 0.1213 | 0.3157 | -7612 | 1 | C=T,T=C |
| rs10220322 | chr14:101130334 | (T/G) | 0.1213 | 0.3157 | -7854 | 1 | C=T,T=G |
| rs28527393 | chr14:101130025 | (T/C) | 0.1213 | 0.3157 | -8163 | 1 | C=T,T=C |
| rs2295659 | chr14:101141544 | (G/T) | 0.1262 | 0.3015 | 3356 | 1 | C=G,T=T |
| rs745797 | chr14:101141909 | (G/A) | 0.1262 | 0.3015 | 3721 | 1 | C=G,T=A |
| rs10141715 | chr14:101172687 | (G/T) | 0.0815 | 0.2886 | 34499 | 0.7667 | C=G,T=T |

**Supplementary Table 2.** Association between treatment resistant schizophrenia and schizophrenia polygenic risk score (PRS SCZ), smoking, age, and sex.

|  | **OR (95% CI)** | **Beta (SE)** | **t-value** | **p-value** |
| --- | --- | --- | --- | --- |
| **Schizophrenia PRS** | 1.3769 (1.2077-1.5737) | 0.3198 (0.0675) | 4.740 | 2.13e-6* |
| **Smoking** | 1.4281 (1.1324-1.8026) | 0.3564 (0.1186) | 3.006 | 0.0027* |
| **Age** | 1.0011 (0.9937-1.0086) | 0.0011 (0.0038) | 0.293 | 0.7696 |
| **Sex (male)** | 1.2430 (0.9800-1.578) | 0.2175 (0.1215) | 1.790 | 0.0734 |

* p <0.05. OR = odds ratio. CI = confidence interval. SE = standard error. PRS = polygenic risk score

**Supplementary Figure 1.** Linkage disequilibrium structure of the lead SNP *rs79229764 C>T* among European populations. The lead SNP is marked by the vertical dashed line.


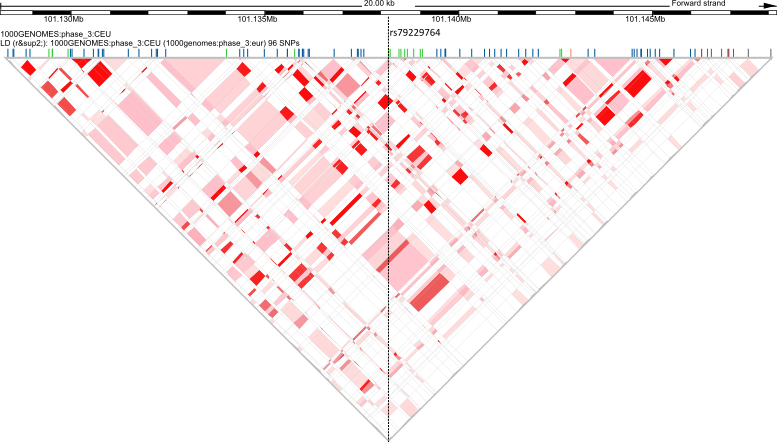

Supplement: Supplementary file 1 — Additional file1 (DOCX 196 KB) [file 40246_2024_673_MOESM1_ESM.docx]
